# Bystander Effects of Nitric Oxide in Cellular Models of Anti-Tumor Photodynamic Therapy

**DOI:** 10.3390/cancers11111674

**Published:** 2019-10-28

**Authors:** Jerzy Bazak, Witold Korytowski, Albert W. Girotti

**Affiliations:** 1 Department of Biophysics, Jagiellonian University, 30-387 Krakow, Poland; jerzy.bazak@gmail.com; 2 Department of Biochemistry, Medical College of Wisconsin, Milwaukee, WI 53226, USA

**Keywords:** photodynamic therapy, bystander effects, nitric oxide, inducible nitric oxide synthase

## Abstract

Tumor cells exposed to stress-inducing radiotherapy or chemotherapy can send signals to non- or minimally exposed bystander cells. Bystander effects of ionizing radiation are well established, but little is known about such effects in non-ionizing photodynamic therapy (PDT). Our previous studies revealed that several cancer cell types upregulate inducible nitric oxide synthase (iNOS) and nitric oxide (NO) after a moderate 5-aminolevulinic acid (ALA)-based PDT challenge. The NO signaled for cell resistance to photokilling as well as greater growth, migration and invasion of surviving cells. Based on this work, we hypothesized that diffusible NO produced by PDT-targeted cells in a tumor might elicit pro-growth/migration responses in non-targeted bystander cells. In the present study, we tested this using a novel approach, in which ALA-PDT-targeted human cancer cells on culture dishes (prostate PC3, breast MDA-MB-231, glioma U87, or melanoma BLM) were initially segregated from non-targeted bystanders via impermeable silicone-rimmed rings. Several hours after LED irradiation, rings were removed, and both cell populations analyzed for various post-hν responses. For a moderate and uniform level of targeted cell killing by PDT (~25%), bystander proliferation and migration were both enhanced. Enhancement correlated with iNOS/NO upregulation in surviving targeted cells in the following order: PC3 > MDA-MB-231 > U87 > BLM. If occurring in an actual tumor PDT setting and not suppressed (e.g., by iNOS activity or transcription inhibitors), then such effects could compromise treatment efficacy or even stimulate disease progression if PDT’s anti-tumor potency is not great enough.

## 1. Introduction

Clinically relevant photodynamic therapy (PDT) was introduced about 45 years ago as a novel means for selectively eradicating a variety of solid tumors via cytotoxic photochemistry [1,2]. As an anti-tumor modality, PDT is unique in requiring three components: a photosensitizing agent (PS), PS-exciting light in the far-visible-to-near-infrared range, and molecular oxygen [3,4,5,6]. PS photoexcitation during PDT gives rise to cytotoxic reactive oxygen species (ROS) such as singlet oxygen (^1^O_2_). The first PS to receive FDA approval for PDT applications was Photofrin^®^, an oligomeric form of hematoporphyrin that continues to be used for a variety of malignancies [4,5,6]. Unlike more conventional chemotherapy or radiotherapy, PDT has few (if any) light-independent side effects and is non-invasive and site-specific, i.e., limited to the tumor upon which light is directed, typically via fiber optic networks [5,6]. Moreover, PDT can often overcome the innate or acquired resistance to other therapies that many tumors exhibit [5]. In addition to pre-existing administered sensitizers such as Photofrin^®^, pro-sensitizers have been developed, one common example being 5-aminolevulinic acid (ALA). Upon entering cancer cells via an amino acid transporter [7], ALA is metabolized to protoporphyrin IX (PpIX), the active PS, via the heme biosynthetic pathway, PpIX accumulating initially in mitochondria [7,8]. For rapid proliferative needs, tumor cells are more active in heme synthesis than normal counterparts [9]. In addition to acting as a PDT sensitizer, ALA-induced PpIX, like that induced by more lipophilic ALA esters, e.g., Hexvix, can serve as a highly effective fluorophore for image-guided tumor resection [10]. Many cancer cells exhibit an intrinsic or acquired resistance to radiotherapy or chemotherapy, and resistance to PDT has also been demonstrated. For ALA-PDT, one notable resistance mechanism involves efflux of newly synthesized PpIX via the plasma membrane transporter ABCG2 [11]. Another important mechanism involves nitric oxide (NO) generated by constitutive and/or PDT stress-upregulated inducible nitric oxide synthase (iNOS/NOS2) [12,13].

NO is a bioactive free radical molecule (1–2 sec lifetime in water) that diffuses freely on its own and, similar to oxygen (O_2_), tends to partition into hydrophobic regions of cells, e.g., cell membranes. At relatively low steady state levels (e.g., 50–300 nM range), NO is known to play a key signaling role in survival, migration, and drug resistance of many different cancer cells [14]. This contrasts with the cytotoxic effects of NO produced at much higher levels (≥1 µM) by activated macrophages during an inflammatory response, e.g., to infection [14,15]. In previous work, we discovered that various cancer cell lines significantly upregulate cytoprotective iNOS and NO after a moderate photodynamic challenge sensitized by ALA-induced PpIX [16,17,18]. The iNOS/NO induction in PDT-surviving (still attached) cells was relatively rapid after irradiation (2–3 h) and prolonged (at least 24 h). Importantly, these cells exhibited a more aggressive growth, migratory, and invasive phenotype than non-photostressed controls, and this was suppressed by inhibition of iNOS or by NO scavenging [19,20,21,22,23]. We postulated that induced diffusible NO from PDT-targeted cells might elicit a pro-growth/migratory response in non- or minimally-targeted bystander cells. In an actual tumor, such targeting diversity might result from uneven PS distribution due to irregularities in the tumor microvasculature [24]. Using photosensitized prostate cancer PC3 cells, we recently observed NO-mediated hyper-aggressive bystander effects for the first time in the context of PDT [25]. In the present study, we compared the ability of four PDT-treated cancer lines (melanoma BLM, glioblastoma U87, breast MDA-MB-231, and prostate PC3) to stimulate NO-dependent aggressiveness in bystander cells of the same type.

## 2. Results

### 2.1. Comparative Upregulation of iNOS/NO in Various Cancer Cell Lines: Responses of ALA/Light-Targeted Cells and Non-Targeted Bystanders

**Figure 1:**
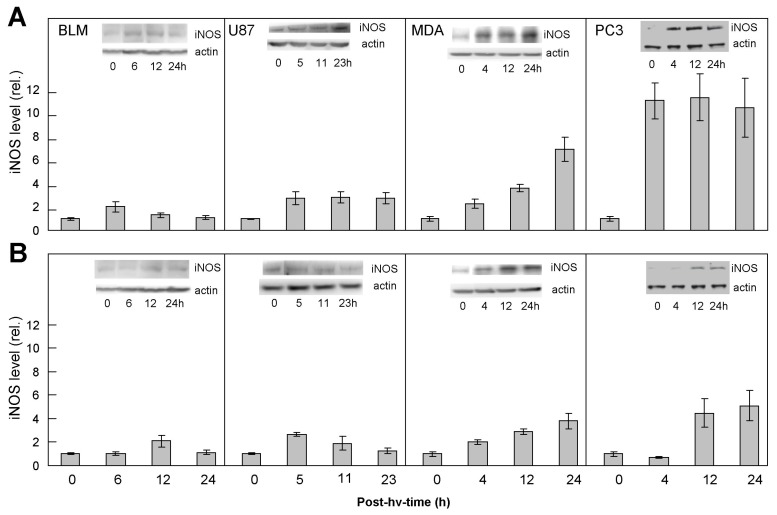
Western blots showing upregulation of iNOS in photodynamically-targeted BLM, U87, MDA-MB-231, and PC-3 cells (**A**) and corresponding bystander cells (**B**). Target cells of each type were incubated for 40 min in the dark with 1 mM 5-aminolevulinic acid (ALA) in medium without serum or phenol red. Cells were then switched to ALA-free medium and exposed to the following light fluences: 0.2 J/cm^2^ (MDA-MB-231 cells); 1.0 J/cm^2^ (BLM, U87, PC3 cells). Bystander cells within two silicone rings were irradiated concurrently. Two hours after irradiation, rings were removed, and the medium switched to 10%-serum-containing RPMI. After the indicated periods of post-irradiation dark incubation, cells from each compartment were recovered for immunoblot analysis of iNOS and β-actin. Cells from two bystander rings were combined for Western analysis. Total cellular protein: 30 µg per lane. Plotted values for each cell line represent iNOS band intensity relative to β-actin and normalized to time-0; means ±SEM (*n* = 2–3). (No difference was observed between time-0 and dark (ALA-only) controls of targeted cells).

Large (13.5 cm) culture dishes were used, each with 2–4 securely attached silicone-rimmed rings. Melanoma BLM, glioblastoma U87, breast MDA-MB-231, or prostate PC3 cells were seeded outside as well as inside the rings and incubated until each population reached 50–60% confluency. At this point, target cells (outside rings) were sensitized with ALA-induced PpIX, after which the entire dish was irradiated using a light fluence that was pre-determined to result in a relatively modest kill for each cell type, i.e., 20–30% at 24 h after irradiation (Table S1). As shown in Figure 1A, all ALA/light-targeted cells exhibited some degree of iNOS induction relative to 0-time samples over a 24 h post-irradiation period, but the extent of induction varied with cell type. For example, during post-hν. incubation, the iNOS level was elevated ~2-fold (at 6 h) in BLM cells, 2–3-fold (at 5 h) in U87 cells, 4–5-fold (at 24 h) in MDA-MB-231 cells, and 8–10-fold (at 24 h) in PC3 cells. Thus, for a set cytotoxic end-point, the increases in iNOS level for these cell lines varied as follows: PC3 > MDA-MB-231 > U87 > BLM. Light exposure without ALA or ALA without light had no significant effect on iNOS level in any of the cell lines used (Figure S1). When non-ALA-exposed bystander cells of each type were examined after irradiation, we again observed an increase in iNOS level (Figure 1B), and its magnitude varied with cell type in approximately the same fashion as seen with the respective targeted cells. In a previous study [25], upregulation of iNOS in bystander PC3 cells was strongly suppressed by an inhibitor of iNOS activity (1400 W) or by a NO scavenger (cPTIO) [26]. It is likely that these inhibitors would have acted similarly on the other cells represented in Figure 1B. This implies that NO generated by targeted cell iNOS played a key role in the observed elevations of bystander iNOS. As shown by the data in Figure 1A, the extent of these elevations depended on the magnitude of iNOS/NO induction in the different PDT-targeted cells.

### 2.2. Comparative Proliferation of the Various Targeted Cells and Their Corresponding Bystanders

**Figure 2:**
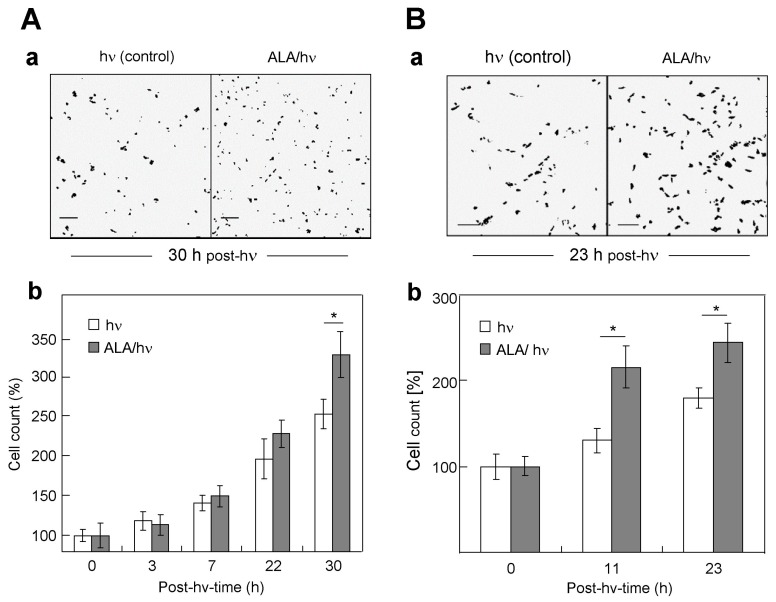
Accelerated proliferation of surviving ALA/light-targeted MDA-MB-231 cells (**A**) and bystander counterparts (**B**). ALA-treated cells and non-treated bystanders were irradiated as described in Figure 1, after which surviving (still attached) ALA/light-challenged cells, now in 10% serum-containing medium, were monitored for proliferation rate compared with light-only (hν) controls. (**a**) Bright-field microscopic images of targeted cells and controls 30 h after irradiation; each bar represents 500 µm; (**b**) Plot of targeted and control cell counts determined by Image-J analysis of microscopic images as in panel (**a**); * *p* < 0.01 compared with light-only controls. (**B**) Bystander responses: (**a**) bright-field images 23 h after irradiation; (**b**) plot of cell counts assessed by Image-J analysis over 23 h of post-hν incubation; each bar represents 500 µm. Plotted values in (**A**) and (**B**) are means ±SEM (*n* = 3); * *p* < 0.01 vs. light-only controls.

Knowing that the signaling activity of endogenous NO can promote the proliferative ability of many cancer cells [27,28,29,30,31,32], we asked whether this would occur after an ALA/light challenge and if so, how the four lines studied might differ in this respect. For the targeted population of MDA-MB-231 cells that survived the challenge, we observed a progressive increase in cell count relative to ALA-only or light-only controls over a 30 h post-irradiation period (Figure 2A). At 30 h, the target cell count was ~30% greater than that of control cells (Figure 2A, panels a and b). The similar growth spurt observed previously for these cells^22^ was strongly attenuated by iNOS inhibitor 1400 W, implicating pro-growth iNOS/NO signaling. For the bystander MDA-MB-231 population from this experiment, we observed a striking increase in growth rate of these cells compared with controls not exposed to ALA/light-treated cells (Figure 2B). For example, at 23 h after irradiation, the bystander count was ~36% greater than that of control cells (Figure 2B, panels a and b). This is the first reported evidence for a pro-growth bystander effect in photodynamically-challenged MDA-MB-231 cells.

As with MDA-MB-231 cells, targeted PC3, U87, and BLM cells that survived the challenge also exhibited a growth spurt compared with non-targeted controls, although the BLM response was insignificantly small. Similarly, the growth rate of bystander cells was greater in each case, except for BLM cells (Figure S2, left panels). However, there was a clear gradation in the magnitude of these responses, which followed the same general trend as observed for the extent of iNOS upregulation (Figure 1).

### 2.3. Comparative Migration of Targeted Cells and Their Corresponding Bystanders

**Figure 3:**
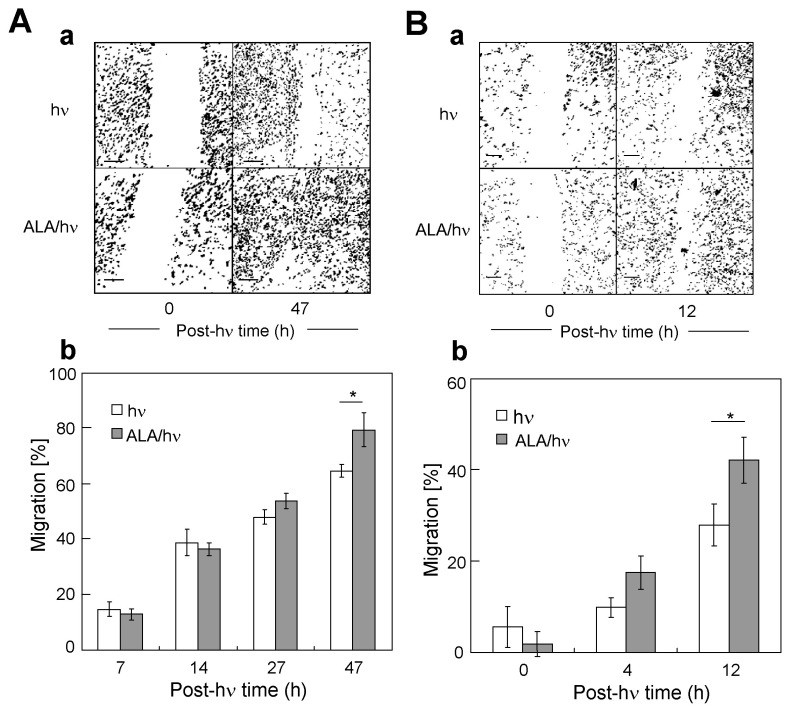
(**A**) Enhanced migration of surviving ALA/light-targeted MDA-MB-231 cells. Immediately after irradiation as described in Figure 1, cells were switched to serum-containing medium, and a linear scratch was made across a selected region of the targeted cell population. The span of the resulting gap was then then monitored over a 2-day dark incubation period. Controls (hν) were monitored alongside. (**a**) Photographs of targeted cell gap zones obtained at 0 h and 47 h after irradiation. (**b**) Extent of targeted cell migration as a function of post-irradiation time. (**B**) Enhanced migration of MDA-MB-231 bystander cells after target cell ALA/light treatment. Immediately after irradiation, separating rings were removed and a linear scratch was made across each bystander group, followed by evaluation of the resulting gap size over a 12 h incubation period. (**a**) Photographs of gap zones at 0 h and 12 h after target cell challenge. (**b**) Plot of bystander migration as a function of post-hν time. Plotted values in (**A**) and (**B**) are means ± SEM (*n* = 5); * *p* < 0.05 vs. light-only controls.

In addition to proliferating more rapidly than non-stressed controls, ALA/light-stressed MDA-MB-231 cells were found to migrate more rapidly, as determined by a gap-closure assay. Thus, photo-stressed cells migrated into a scratch-voided (gap) zone more rapidly than non-stressed controls; at 47 h post-hν, for example, ~25% more of the former had moved into the gap zone (Figure 3A). Bystander cells from the same experiment behaved similarly. For example, at 4 h and 12 h post-hν, bystander migration into the gap area exceeded that of control cells by ~70% and ~56%, respectively (Figure 3B). As with the observed pro-growth effect, this is the first evidence for a pro-migration effect on bystander MDA-MB-231 cells in a PDT-like setting.

As anticipated from the graded post-hν growth spurts of PC3, U87, and BLM bystander cells, graded migration spurts were observed in these cells as well (Figure S2, right panels). Once again, the general trend was comparable to that seen for targeted iNOS induction in the different cell lines (Figure 1).

### 2.4. Effects of an iNOS Inhibitor and NO Scavenger on Proliferation/Migration of Bystander Cells

**Figure 4:**
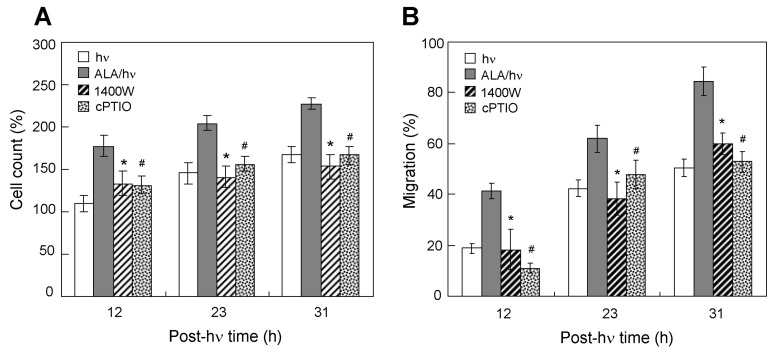
Effects of an iNOS inhibitor or NO scavenger on proliferation and migration of MDA-MB-231 bystander cells exposed to ALA/light-targeted counterparts. After sensitization with ALA-induced PpIX, target cells (separated from ring-enclosed bystanders) were switched to fresh medium without ALA, then exposed to a light fluence of 0.2 J/cm^2^ as described in Figure 3. A non-ALA-treated control was irradiated alongside. After a 1 h dark period, the separating rings were removed, and cells were switched to 10% serum-containing medium. Where indicated, 25 µM 1400 W (W) or 25 µM cPTIO (cP) was included in both cell compartments before irradiation and maintained at this concentration during subsequent dark incubation. (**A**) Bystander proliferation during post-irradiation incubation of both cell populations. Cell counts were determined via Image-J analysis; plotted values are means ±SEM (*n* = 6); (**B**) Bystander migration during post-irradiation incubation, as determined by gap-closure assay; means ±SEM (*n* = 5). (**A**) and (**B**): * *p* < 0.05 vs. ALA/hν; ^#^ *p* < 0.05 vs. ALA/hν.

In previous work, we established that iNOS-derived NO played a dominant role in the accelerated growth and migration of surviving ALA/light-challenged cancer cells by showing that these responses were strongly attenuated by iNOS inhibitor 1400 W or NO scavenger cPTIO. The same agents were used in the present study to determine whether targeted cell iNOS/NO was necessary for driving the pro-growth/migration bystander effects described. As shown in Figure 4A, when 1400 W was introduced prior to irradiation of MDA-MB-231 target cells, the otherwise accelerated growth of non-contacting bystander cells (Figure 2B) was sharply reduced, e.g., by ~35% at 31 h post-hν Likewise, when cPTIO (a highly selective NO scavenger [26] was present, the bystander growth rate was again blunted (Figure 4A)—in this case by ~26% at 31 h post-hν As anticipated, the accelerated migration of MDA-MB-231 bystander cells in response to ALA/light-challenged counterparts was also attenuated by 1400W and cPTIO. For example, faster migration at 31 h post-hν was inhibited ~30% by 1400 W and ~38% by cPTIO (Figure 4B). These agents had similar inhibitory effects on augmented proliferation and migration of U87, PC3, and BLM bystander cells. Collectively, these findings indicate that NO generated by targeted cell iNOS played a key driving role in the acquired greater proliferative and migratory aggressiveness of bystander cells. In a previous study, further evidence for iNOS/NO involvement was obtained by showing that iNOS knockdown in target PC3 cells prevented bystander cells from becoming more aggressive [17]. Therefore, diffusible NO from targeted cell iNOS clearly played a major role in the acquired bystander responses.

### 2.5. Stimulation of Bystander Cell Growth and Migration with a Chemical NO Donor

**Figure 5:**
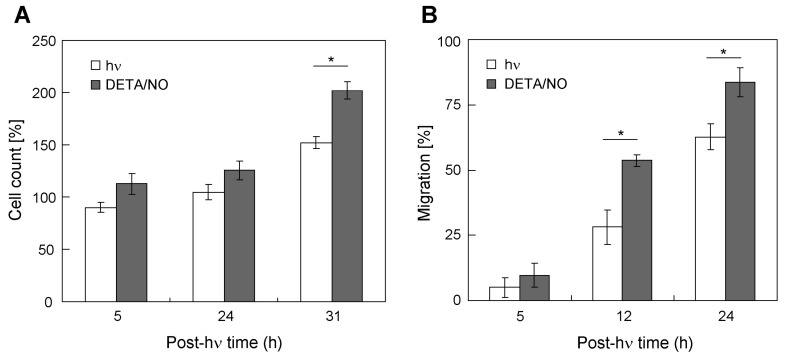
Effects of a chemical NO donor on proliferation and migration of MDA-MB-231 cells. (**A**) Proliferation. Cells at ~40% confluency in 10% serum-containing RPMI medium were incubated in the absence (open bars) or presence (filled bars) of DETA/NO at a starting concentration of 50 µM. Cell counts were determined by Image-J analysis of photomicrographs acquired after the indicated incubation times. Plotted data are means ±SEM (*n* = 3); * *p* < 0.05 vs. control. (**B**) Migration. Cells in 10% serum-RPMI were incubated in the absence (open bars) or presence (filled bars) of 10 µM DETA/NO, and at the indicated times evaluated for migration by gap-closure assay. Data are means ±SEM (*n* = 3); * *p* < 0.01 vs. control.

To obtain more direct evidence for NO involvement in target cell-stimulated bystander growth/migration, we exposed cells to a NONOate chemical donor, DETA/NO. DETA/NO spontaneously decomposes to release 2 NO molecules with a half-life of ~20 h at 37 °C in aqueous solution at pH 7.4 [33]. As shown in Figure S3 and Figure 5A), DETA/NO at a starting concentration of 50 µM stimulated MDA-MB-231 cell proliferation over a time period amounting to ~1.5-times the donor half-life. The cell count during DETA/NO treatment was consistently greater than that of non-treated controls, reaching ~33% greater after 31 h. As shown in Figure 5B, DETA/NO also caused a significant increase in cell migration over that of controls, the starting concentration in this case being 10 µM. For example, with the donor present, cell migration was ~30% greater after 24 h. No significant stimulation of proliferation or migration was observed when fully decomposed DETA/NO was used, ruling out any non-specific effects of the donor itself. The results described in this section provide additional support for our hypothesis that NO diffusing from upregulated iNOS in targeted cells played a key signaling role in the stimulation of bystander aggressiveness.

### 2.6. Evaluating Possible Effects of Conditioned Media on Bystander Cells

**Figure 6:**
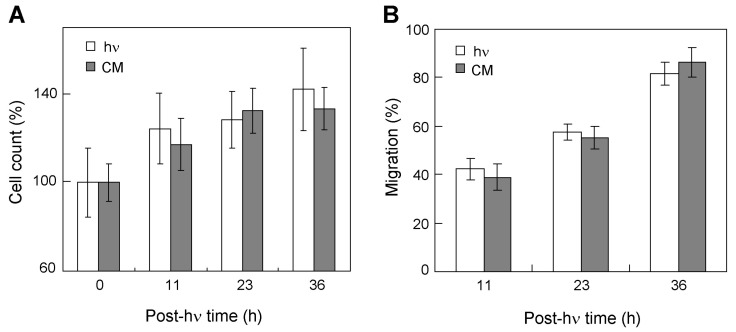
Proliferation and migration of non-stressed MDA-MB-231 bystanders after exposure to conditioned medium from photodynamically-stressed counterparts. After preincubation with ALA in serum-free medium, target cells (separated from ring-enclosed bystanders) were irradiated (0.2 J/cm^2^). After this, cells in both compartments were switched to 10% serum-containing medium and dishes were returned to the incubator. Six hours later, the bystander medium was removed and replaced with conditioned medium (CM) from photodynamically targeted cells or from non-targeted controls (hν), after which cell growth and migration were evaluated. Plotted data are means ±SEM (*n* = 6).

We asked whether other diffusible factors besides NO might contribute to the bystander effects described. Such factors would have to be relatively long-lived or, as NO, generated continuously by targeted cells. Species longer lived than NO might include cytokines such as TNF-α or TNF-β, NADPH oxidase (NOX)-generated H_2_O_2_, or membrane lipid-derived hydroperoxides [34,35,36]. To study this possibility, we carried out a typical target-bystander experiment on MDA-MB-231 cells using non-challenged bystander cells inside rings and ALA/light-challenged cells outside. After 6 h of post-hν dark incubation, the medium was replaced with conditioned medium from targeted cells. During subsequent incubation, we tracked bystander cell proliferation and found that there was no significant change relative to the control (Figure 6A). Likewise, conditioned medium from ALA/light-treated MDA-MB-231 cells had no significant effect on the migration rate of bystander counterparts (Figure 6B). This agrees with previous findings on PC3 cells treated with conditioned medium [25]. Thus, there was consistency between the two different cancer lines studied. By implication, long-lived mobile species were ruled out as contributors to bystander effects described for all four lines in this study, and NO appeared to be the predominant signaling mediator.

### 2.7. Correlation between Enhanced Proliferation/Migration and iNOS Elevation for a Given Extent of Cell Kill

**Figure 7:**
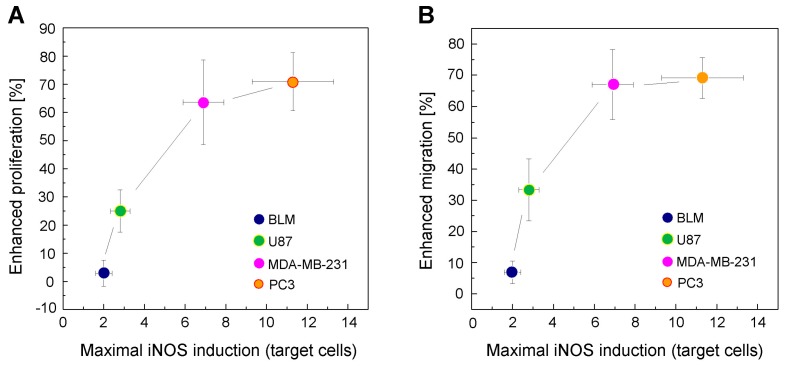
Increased proliferation and migration of BLM, MDA-MB-231, U87, and PC3 bystander cells as a function of maximal iNOS induction in targeted counterparts. (**A**) Elevated proliferative potency vs. greatest extent of targeted iNOS upregulation for the different cell lines. (**B**) Elevated migratory potency vs. greatest extent of targeted iNOS upregulation for the different lines. Data points for proliferation or migration are means ±SEM (*n* = 5–6) and those for iNOS are means ±SEM (*n* = 3).

Using 20–30% photokilling as a pre-set reference point for each of the four cancer lines studied, we asked how bystander aggressiveness would correlate with extent of iNOS induction in targeted cells. As shown in Figure 7A, there was an approximately linear increase in bystander hyper-proliferation with iNOS upregulation, BLM cells showing the smallest response and PC3 cells the greatest. A similar trend was observed for bystander hyper-migration, BLM cells with little iNOS induction showing the weakest response and PC3 cells, with the greatest induction, showing the strongest response (Figure 7B). Thus, the extent of iNOS/NO upregulation in PDT-targeted cells was a direct determinant of enhanced bystander proliferation/migration aggressiveness in the following decreasing order: PC3 > MDA-MB-231 > U87 > BLM.

## 3. Discussion

It is well established, particularly in the realm of therapeutic ionizing radiation, that not all cells in a given tumor will be targeted uniformly, but that well-exposed cells can send stress signals to unexposed or poorly exposed neighbors, i.e., bystander cells [37,38,39]. X-rays, γ-rays, or α-particles can elicit a variety of responses in bystander cells ranging from DNA damage, impaired damage repair and apoptotic cell death to accelerated proliferation and motility [37]. Cells directly targeted by radiation can send signals to non-targeted bystanders by at least two different means: (i) via gap junctions between cells or (ii) via the medium without actual cell contact [37,38]. There has been increasing evidence in support of the latter mechanism as methods for assessing it have improved. A variety of signaling molecules capable of traversing aqueous media from radiation-targeted cells to bystanders have been identified, including (i) cytokines such as TGF-β and TNF [40,41], (ii) ROS such as hydrogen peroxide (H_2_O_2_) [42,43], and (iii) NO or NO-derived species [44,45,46]. Like O_2_, NO diffuses rapidly, and is attracted to hydrophobic sites such as cell membranes [30,31,32]. Unlike H_2_O_2_ with catalase or glutathione peroxidase, NO is not susceptible to any known specific enzymatic scavenging. However, NO has a short half-life in aqueous media (1–2 sec) [47], requiring its continuous generation by targeted cells in order to induce bystander effects.

The possibility of bystander effects in conjunction with non-ionizing PDT has been recognized for about 20 years [34,35], but far less is known about this in mechanistic terms than for its ionizing radiation counterpart. In an earlier initial study on the possible role of NO in ALA/light-induced bystander effects, we used a novel silicone ring approach for segregating targeted PC3 cells from non-targeted bystanders [25]. The rings were removed after a suitable post-irradiation interval, allowing any diffusible stress-upregulated factors to flow from the targeted to bystander zones. As expected from earlier work on ALA/light-challenged PC3 cells, targeted cells exhibited progressive upregulation of iNOS/NO with more rapid NO-dependent growth and migration of surviving cells [25]. Strikingly similar responses (upregulation of iNOS/NO, faster growth/migration) were observed in bystander cells and were found to be dependent on primary NO from targeted cells [25]. In addition to iNOS in PC3 bystanders, several other pro-survival/expansion effectors were upregulated in 1400 W- or cPTIO-inhibitable manner, including cyclooxygenase-2 (COX-2) and protein kinases Akt and ERK1/2 [25]. This was the first reported evidence for NO-dependent bystander effects in the context of PDT and raised serious questions about the tumor-promoting potential of such effects in clinical PDT settings. 

In the present study, we compared four human cancer lines (breast MDA-MB-231, prostate PC3, glioma U87 and melanoma BLM) for pro-growth/migration bystander effects of NO induced by targeted photodynamic stress. ALA treatment was the same for all lines, but fluences for subsequent light exposure were varied such that a consistent targeted cell kill was obtained, i.e., ~25% at 24 h after irradiation. Under these conditions, surviving cells exhibited varying extents of iNOS protein upregulation, i.e., PC3 > MDA-MB-231 > U87 > BLM. The same order of iNOS induction was observed for bystander cells, although this induction (e.g., in PC3 cells) occurred more slowly than that which occurred in targeted counterparts during post-hν incubation. In addition to iNOS upregulation, there was a striking functional consequence of targeted cell iNOS/NO upregulation on bystander cells, viz. accelerated proliferation and migration, which followed the same general order as observed for iNOS upregulation. Thus, BLM cells with the smallest induction showed the smallest increase in bystander proliferation/migration while MDA-MB-231, U87 and PC3 cells with increasingly greater inductions showed correspondingly greater proliferation/migration. These novel findings, along with the non-effects of conditioned media from targeted cells, highlight the importance of targeted cell iNOS/NO in stimulating bystander aggressiveness. Our findings suggest that a type of relay process is set in motion during a photodynamic challenge whereby NO initially overproduced by targeted cells diffuses to unaffected bystanders and induces iNOS/NO there, thus beginning a NO-mediated process that propagates through the bystander population. A similar NO-relay process may occur in any photosensitized cell population, including any of those we described as “targeted”. However, it was only through our ability to separate and differentiate bystander and targeted cells that NO-mediated bystander effects could be detected and evaluated. When observed using ionizing radiation, this process has been described as a “feed-forward field effect” of NO, suggesting a far-reaching self-propagation phenomenon [48].

As anticipated from our in vitro observations on bystander cells, accelerated growth and migration of such cells in an actual tumor after PDT might promote tumor growth and metastatic expansion. This possibility should raise concerns because tumor cells not lethally damaged by PDT might send dangerous NO-mediated pro-expansion signals to non- or minimally-stressed bystanders. As predicted from this study, the extent of such signaling can vary with the extent of PDT-induced iNOS. For clinical PDT, these negative side effects might be minimized through pharmacologic use of iNOS enzymatic inhibitors such as L-NIL and GW274150, which have already tested safely in clinical trials unrelated to PDT [49,50]. Another promising agent is bromodomain and extra-terminal (BET) inhibitor JQ1, which strongly suppresses cancer persistence and progression in vivo [51], and was recently shown to prevent iNOS transcription in ALA/light-challenged glioblastoma cells [51].

## 4. Materials and Methods

### 4.1. General Materials

Cell culture media (RPMI, DMEM, DMEM/F12 (1:1),), fetal bovine serum (FBS), antibiotics (streptomycin, penicillin), L-glutamine, and 5-aminolevulinic acid (ALA) were obtained from Sigma-Aldrich (St. Louis, MO, USA). Cayman Chemicals (Ann Arbor, MI, USA) supplied the following reagents: (3-(4,5-dimethylthiazol-2-yl)-2,5-diphenyltetrazolium bromide (MTT), N-[3 (aminomethyl) benzyl] acetamidine (1400W), 2-(4-carboxyphenyl)-4,4,5,5-tetramethylimidazolone-1-oxyl-3-oxide (cPTIO), DETA-NONOate (DETA/NO), and a polyclonal antibody against human iNOS. A rabbit monoclonal antibody against human β-actin and horseradish peroxidase-conjugated IgG secondary antibodies were from Cell Signaling Technologies (Danvers, MA). Pierce chemical Co. (Rockford, IL) supplied reagents for bicinchronic (BCA) protein assays and for SuperSignal West Pico chemiluminescence detection of proteins on Western blots. The silicone flexi-Perm^®^ micro-12 inserts and flexi-Perm-ConA^®^ rings for separating targeted cells from bystanders were obtained from Sarstedt-AG (Numbrecht, Germany).

### 4.2. Cell Culture Conditions

Human breast cancer MDA-MB-231, glioblastoma U87, and prostate cancer PC3 cells were obtained from and authenticated by the American Type Culture Collection (Manassas, VA, USA). Human melanoma BLM cells derived from lung metastases of skin melanoma were obtained as a research gift from Dr. G.N.P. van Muijen ((Radboud University, Nijmegen, The Netherlands)).

### 4.3. Segregation of Targeted Cells from Bystanders

Each cell type was grown in a culture dish (85-mm diameter) (Sigma-Aldrich, Poznan, Poland) onto which a 12-well open-bottom silicone retainer (flexi-PERM^®^ micro-12, Sarstedt, Numbrecht, Germany) was firmly attached. Each circular well of the retainer had a bottom diameter of 6.2 mm. Cells intended for photodynamic targeting were seeded outside the 12-well insert while those intended as bystanders were seeded inside the wells. Seeding densities for each cell type were as follows. For proliferation experiments: ~2 × 10^3^ bystander cells per well in 0.1 mL of growth medium; for migration experiments: ~4 × 10^3^ bystander cells per well in 0.1 mL of medium. Approximately 1 × 10^6^ target cells in 10 mL of growth medium were used in each case. No initial contact was made between media in the target cell and bystander populations.

For recovering sufficiently large amounts of cellular protein for iNOS immunoblots, a different arrangement was used. In this case, cells were grown in a 135-mm dish (Sigma-Aldrich, Poznan, Poland) onto which 2–4 silicone-rimmed flexi-Perm-ConA^®^ rings (12-mm bottom inner diameter, Sarstedt, Numbrecht, Germany) were attached. Cells outside the rings were photodynamically targeted while those inside the rings acted as bystanders. Seeding densities for each cell type were as follows: ~3 × 10^6^ target cells in 15 mL growth medium and ~5 × 10^3^ bystander cells per ring in 1 mL of medium. Once again, the media in the two cell populations made no contact with each other at this stage.

### 4.4. Cell Photosensitization and Irradiation

When target cells reached ~65% confluency after 24 h, they were metabolically sensitized with PpIX by treating with 1.0 mM ALA in serum- and phenol red-free RPMI medium and returning to the incubator for 40 min. At this point, PpIX was localized primarily in cellular mitochondria, as revealed by fluorescence microscopy [21]. No significant fluorescence was observed in bystander cells, confirming that ALA-induced PpIX was limited to targeted cells. The medium was then switched to fresh RPMI lacking ALA; where indicated, 1400 W or cPTIO was included, the final concentration of either agent being 25 µM. Immediately after ALA treatment (−/+ 1400 W or cPTIO), dishes were placed on a flat-surface light diffuser over a broad-band visible LED source. The irradiance (fluence rate) of this source was ~1.1 mW/cm^2^, as measured with a Model ILT-1400A radiometer (International Light Technologies, Peabody, MA, USA) with probe positioned on the bottom surface of an empty culture dish. Light doses (fluences) were either 0.2 J/cm^2^ (MDA-MB-231 cells) or 1 J/cm^2^ (BLM, U87, PC3 cells), which caused the same amount of killing (~25%) for the different cell lines. A fluence of 1 J/cm^2^ corresponded to an irradiation time of 15 min. Irradiation was carried out at room temperature, which remained at 25 ± 2 °C at the culture dish surface. Immediately thereafter, the medium of targeted and bystander cells was aspirated and replaced with 10% FBS in RPMI medium. After ~2 h of dark incubation, the medium was aspirated from both compartments and the silicone retainers were carefully removed. Immediately thereafter, all cells were overlaid with 10% FBS in RPMI. During subsequent dark incubation for increasing times up to 48 h, cell proliferation and migration rates were determined as described below. Target cells treated with ALA alone or light alone were analyzed alongside as controls. Other details were as previously described [20,21,25].

### 4.5. Assessment of Targeted Cell Photokilling

The effects of an ALA/light challenge on overall cell viability was assessed by MTT assay, which was typically carried out 24 h after irradiation. Assay conditions were as described previously [19,20].

### 4.6. Western Blot Analyses

Levels of iNOS protein in ALA/light-targeted cells and bystander counterparts were monitored by Western blotting. The silicone ring-based approach was used for these determinations because total protein recovery was inadequate using the 12-well retainer approach. Cell recovery from the targeted and bystander areas was accomplished by careful scraping, as described [25]. After cell lysis and protein determination [25], samples of equal protein concentration were subjected to Laemmli SDS-PAGE, using NuPAGE 3–8% acrylamide/bis-acrylamide for analyzing iNOS (monomer mass ~130 KDa). After separation, proteins were electrophoretically transferred to a PVDF membrane. After incubation with an iNOS primary antibody, the membrane was treated with a peroxidase-conjugated IgG secondary antibody. Protein bands were visualized using the Supersignal West Pico chemiluminescence system and quantified using ImageJ-1.49v [25]. β-Actin was also quantified as a loading standard.

### 4.7. Determination of Targeted and Bystander Cell Proliferation

Photomicrographs of surviving ALA/light-treated cells (−/+ 1400 W) and corresponding bystanders were taken at various times after irradiation. Controls (ALA-only or light-only target cells) were photographed alongside at the same time points. After the photos were scanned, an online Image-J program was used to obtain cell counts for targeted and bystander cells relative to their controls. At least six different scanning zones were evaluated for each timed target/bystander population pair. Data from at least three replicate dishes were pooled and expressed as means ±SEM.

### 4.8. Determination of Targeted and Bystander Cell Migration

A “wound-healing” or gap-closure assay was used to assess the effects of an ALA/light challenge (−/+ 1400 W) on the migration of surviving targeted cells and their bystanders. At ~2 h after irradiation, the 12-well insert separating the cell populations was removed and a linear scratch was made across each at some distinct site using a sterile 200 µL pipette tip. After incubation for various periods up to 48 h, cells in the gap area were photographed using a Nikon Eclipse TS100 phase contrast microscope equipped with a Mitocam Pro-282B camera. Extent of bystander cell migration (gap closure) compared with that of a light-only or ALA-only control was determined by analysis of representative images at each post-irradiation time. Percent gap closure was calculated according to the following expression: 100× [(time-0 gap span)–(time-t gap span)/(time-0 gap span)]. Data were obtained from at least six replicate experiments for each reaction condition.

### 4.9. Bystander Responses to Conditioned Media from Targeted Cells

Target MDA-MB-231 cells were treated with ALA and exposed to a light fluence of 0.2 J/cm^2^. After 6 h of dark incubation, the medium was collected, centrifuged, and transferred to another dish with fresh bystander cells inside rings. Bystander counts and migration were then determined after various incubation times. Control bystanders treated with medium from ALA-treated, but not irradiated target cells were incubated and analyzed alongside. Data from at least three replicate experiments were evaluated.

### 4.10. Statistical Evaluations

A two-way analysis of variance (ANOVA) was used to determine if differences existed in experimental values, followed by a Tukey-Kramer HSD test to identify which values were different or a Student’s *t*-test. Plotted values are presented as means ±SEM of data from at least three separate experiments. Perceived differences in experimental values were considered statistically significant if *p* values were <0.05.

## 5. Conclusions

This study advances our knowledge of NO-mediated bystander effects by comparing four different human cancer lines for sensitivity to such effects initiated by ALA-PDT-targeted cells of each type. The order of accelerated bystander proliferation and migration after targeted cell photochallenge paralleled the extent of iNOS/NO upregulation in the latter, prostate PC3 cells showing the greatest responses and melanoma BLM the smallest. For solid tumors subjected to PDT, uneven distribution of photosensitizing agents and exciting light would open possibilities for bystander effects, allowing upregulated NO from targeted cells to elicit more iNOS/NO and greater aggressiveness in non-targeted bystanders, thereby producing a NO-feed forward phenomenon. Pharmacological inhibitors of iNOS activity or iNOS expression are advocated to suppress these negative effects of PDT.

## Supplementary Materials

The following are available online at https://www.mdpi.com/2072-6694/11/11/1674/s1, Figure S1: Western blots showing iNOS status in light-only controls (LOC) and ALA-only controls (AOC); Figure S2: Proliferation and migration of U87, BLM, and PC3 bystander cells in response to target cell photodynamic stress; Figure S3: Effects of a chemical NO donor (DETA/NO) on proliferation of MDA-MB-231 cells;, Table S1: Cell viability as a function of light dose for ALA-PDT.
