# Supplementary material for: Bystander Effects of Nitric Oxide in Cellular Models of Anti-Tumor Photodynamic Therapy
Source: Cancers (Basel). 2019 Oct 28;11(11):1674. doi: 10.3390/cancers11111674 (PMC6895962; doi:10.3390/cancers11111674)
Supplement: Supplementary file 1 [file cancers-11-01674-s001.zip › supplementary-proof/supplementary-final-proof.pdf]

cancers

MDPI

Article

Bystander Effects of Nitric Oxide in Cellular Models of Anti-Tumor Photodynamic Therapy

Jerzy Bazak [1], Witold Korytowski [1,*] and Albert W. Girotti [2,*]

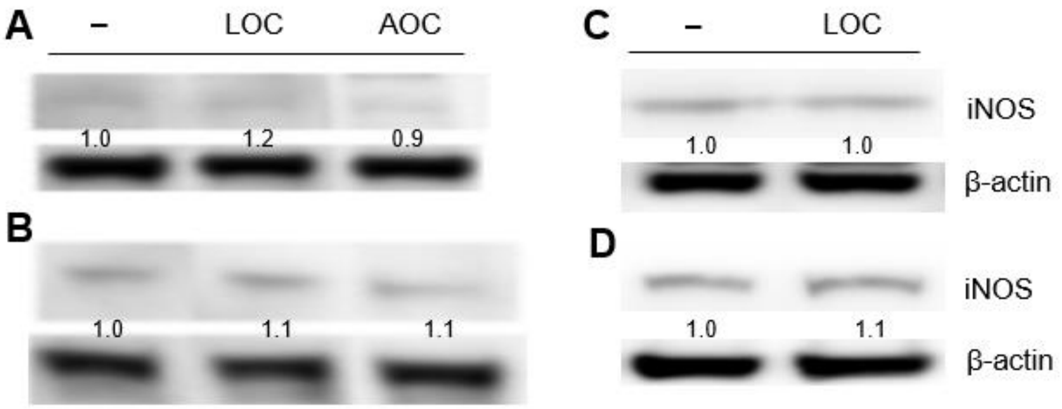

Figure S1. Western blots showing iNOS status in light-only controls (LOC) and ALA-only controls (AOC) of (**A**) PC3, (B) MDA-MB-231, (**C**) U87, and (D) BLM cells; (-) controls with no ALA or light.. Where indicated, cells were either treated with 1 mM ALA or exposed to the following light fluences: 0.2 J/cm^2 (**B**); 1.0 J/cm^2 (A, C, D). Cells were recovered and analysed after 12 h of dark incubation. Total cellular protein: 70 μg per lane. Numbers indicate iNOS band intensity relative to procedural control (-).

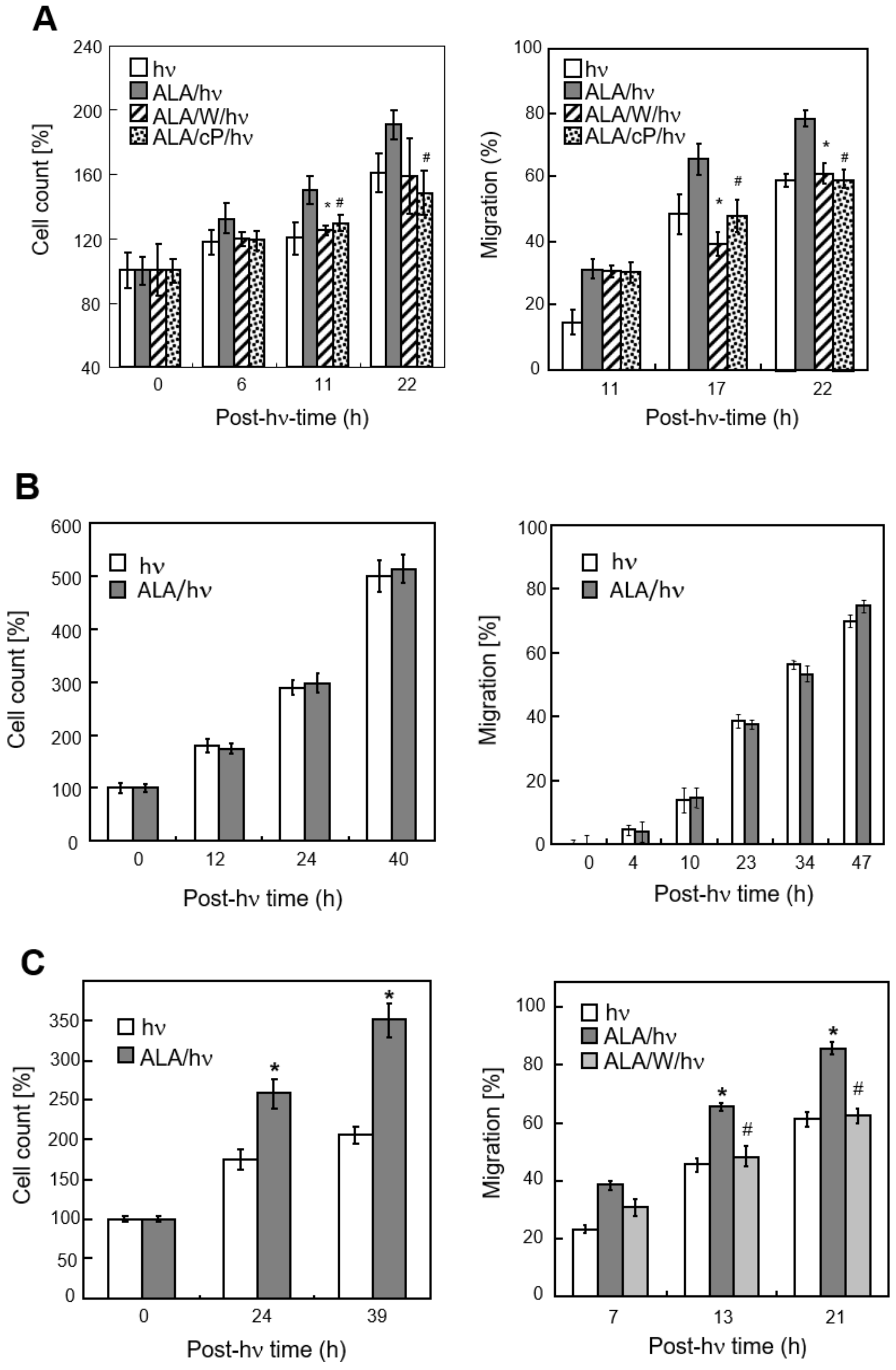

Figure S2. Proliferation (left panels) and migration (right panels) of (**A**) U87, (B) BLM, and (C) PC3 bystander cells in response to target cell photodynamic stress. Target cells in serum-free medium were preincubated with ALA and irradiated (1 J/cm^2). After a 1 h dark period, the separating rings were removed and cells were switched to 10% serum-containing medium lacking or containing 25 μM 1400 W (ALA/W/hν) or cPTIO (ALA/cP/hν), as indicated. Plotted values are means ±SEM (n = 3); (**A**) * $p <$ 0.05 *vs.* ALA/hν; (**C**) * $p < 0.05$ vs. hν, $^{\#}$P<0.05 *vs.* ALA/hν.

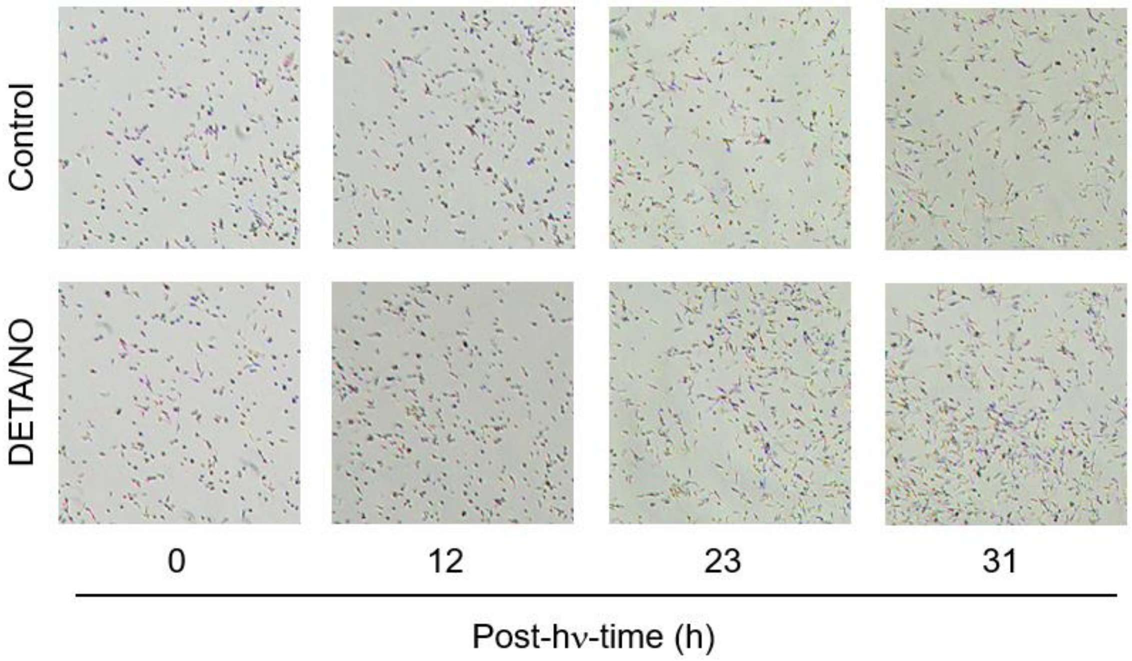

Figure S3. Images showing effects of a chemical NO donor (DETA/NO) on proliferation of MDA-MB-231 cells. Additional details are provided in Figure 5 legend.

Cell lines \ Light fluence (J/cm^2)	0.0	0.2	0.4	0.5	0.6	1.0	1.5	2.0	2.5	3.0
PC3	100 ± 5.0	----	----	93 ± 4.0	----	**82 ± 5.5**	----	53 ± 2.3	----	----
MDA-MB-231	100 ± 4.5	**76 ± 1.3**	69 ± 2.4	63 ± 5.0	58 ± 5.0	40 ± 3.0	----	1 ± 1.0	----	0 ± 2
U87	100 ± 0.8	75 ± 3.6	----	72 ± 2.4	----	**73 ± 2.2**	----	57 ± 0.9	----	27 ± 0.5
BLM	100 ± 5.3	----	----	92 ± 4.9	----	**77 ± 1.3**	71 ± 6.1	68 ± 1.4	59 ± 0.3	----

Table S1. Percent cell viability as a function of light dose for ALA-PDT. Cells from each cancer line at ~60% confluency in serum- and phenol red-free RPMI medium in 35-mm dishes were incubated in the presence of 1 mM ALA for 40 min in the dark. After switching to ALA-free medium, cells were exposed to the indicated fluences of LED light and after 24 h of dark incubation were checked for viability using an MTT-based assay. Means ± SEM of values from three separate determinations on each cell type are shown.
