# Supplementary material for: Bystander Effects of Nitric Oxide in Cellular Models of Anti-Tumor Photodynamic Therapy
Source: Cancers (Basel). 2019 Oct 28;11(11):1674. doi: 10.3390/cancers11111674 (PMC6895962; doi:10.3390/cancers11111674)
Supplement: Supplementary file 1 [file cancers-11-01674-s001.zip › supplementary-proof/supplementary-proof.docx]

Article

Bystander Effects of Nitric Oxide in Cellular Models of Anti-Tumor Photodynamic Therapy

Jerzy Bazak ^1^, Witold Korytowski ^1,^* and Albert W. Girotti ^2,^*


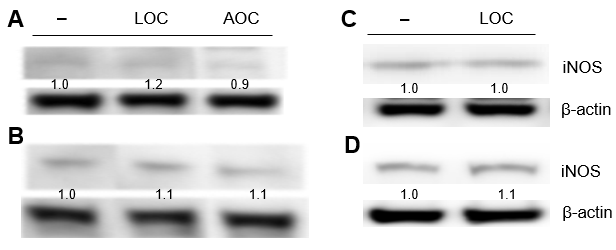


**Supplementary Figure S1.** Western blots showing iNOS status in light-only controls (LOC) and ALA-only controls (AOC) of (**A**) PC3, (B) MDA-MB-231, (**C**) U87, and (D) BLM cells; (-) controls with no ALA or light.. Where indicated, cells were either treated with 1 mM ALA or exposed to the following light fluences: 0.2 J/cm^2^ (**B**); 1.0 J/cm^2^ (A, C, D). Cells were recovered and analysed after 12 h of dark incubation. Total cellular protein: 70 μg per lane. Numbers indicate iNOS band intensity relative to procedural control (-).


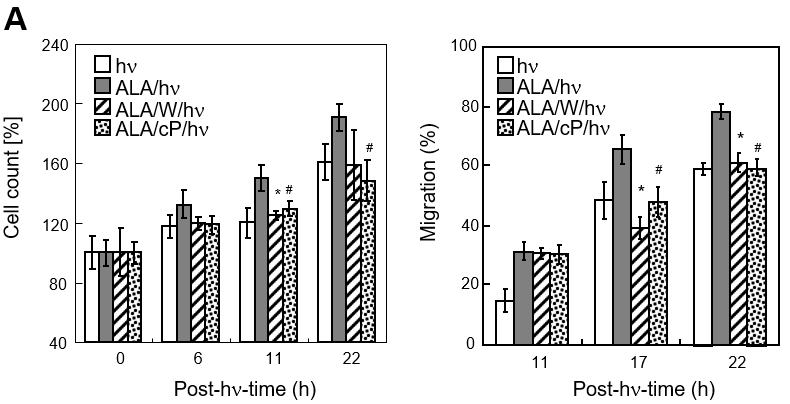

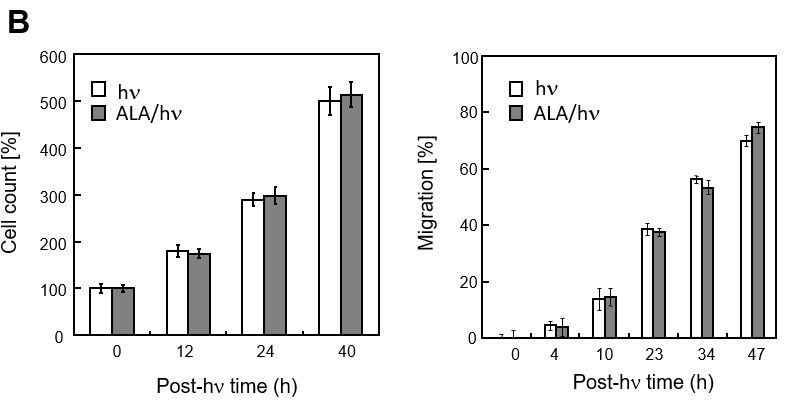

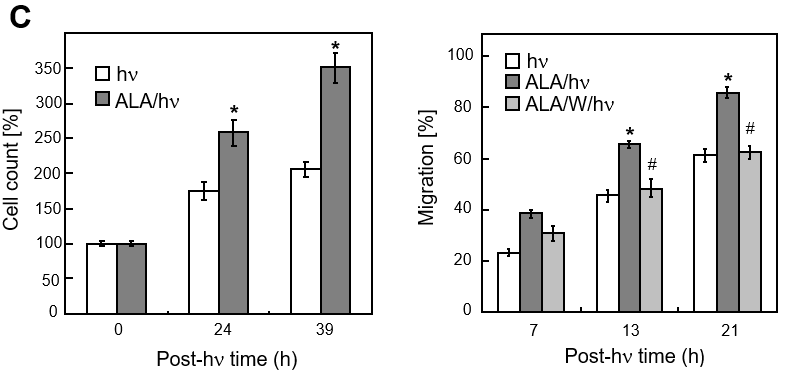


**Supplementary Figure S2.** Proliferation (left panels) and migration (right panels) of (**A**) U87, (B) BLM, and (C) PC3 bystander cells in response to target cell photodynamic stress. Target cells in serum-free medium were preincubated with ALA and irradiated (1 J/cm^2^). After a 1 h dark period, the separating rings were removed and cells were switched to 10% serum-containing medium lacking or containing 25 µM 1400 W (ALA/W/hν) or cPTIO (ALA/cP/hν), as indicated. Plotted values are means ±SEM (*n* = 3); (**A**) * *p* < 0.05 *vs.* ALA/hν; (**C**) * *p* < 0.05 vs*.* hν, ^#^P<0.05 *vs.* ALA/hν.


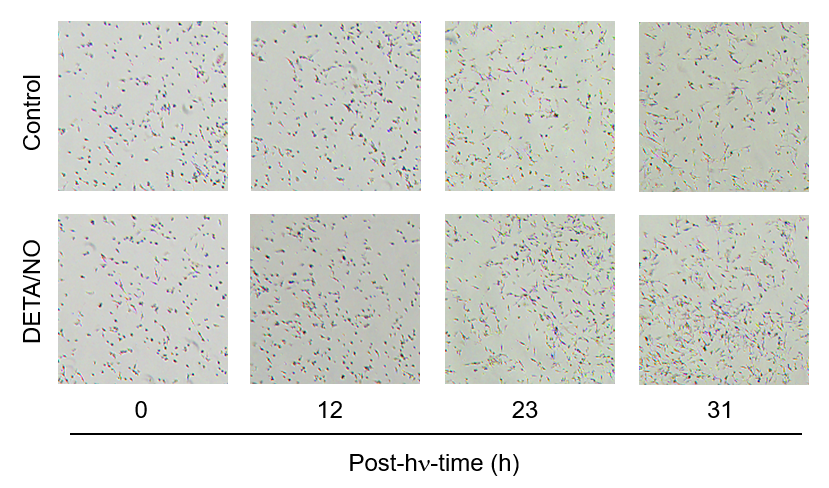


**Supplementary Figure S3.** Images showing effects of a chemical NO donor (DETA/NO) on proliferation of MDA-MB-231 cells. Additional details are provided in Figure 5 legend.


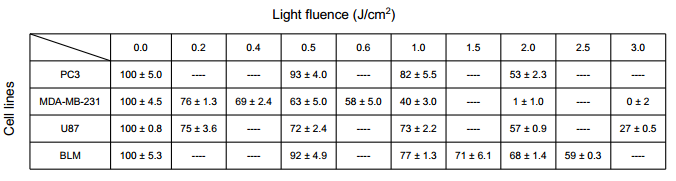


**Supplementary Table S1.** Percent cell viability as a function of light dose for ALA-PDT. Cells from each cancer line at ~60% confluency in serum- and phenol red-free RPMI medium in 35-mm dishes were incubated in the presence of 1 mM ALA for 40 min in the dark. After switching to ALA-free medium, cells were exposed to the indicated fluences of LED light and after 24 h of dark incubation were checked for viability using an MTT-based assay. Means ± SEM of values from three separate determinations on each cell type are shown.
