# Supplementary figures and images for: Bystander Effects of Nitric Oxide in Cellular Models of Anti-Tumor Photodynamic Therapy
Source: Cancers (Basel). 2019 Oct 28;11(11):1674. doi: 10.3390/cancers11111674 (PMC6895962; doi:10.3390/cancers11111674)

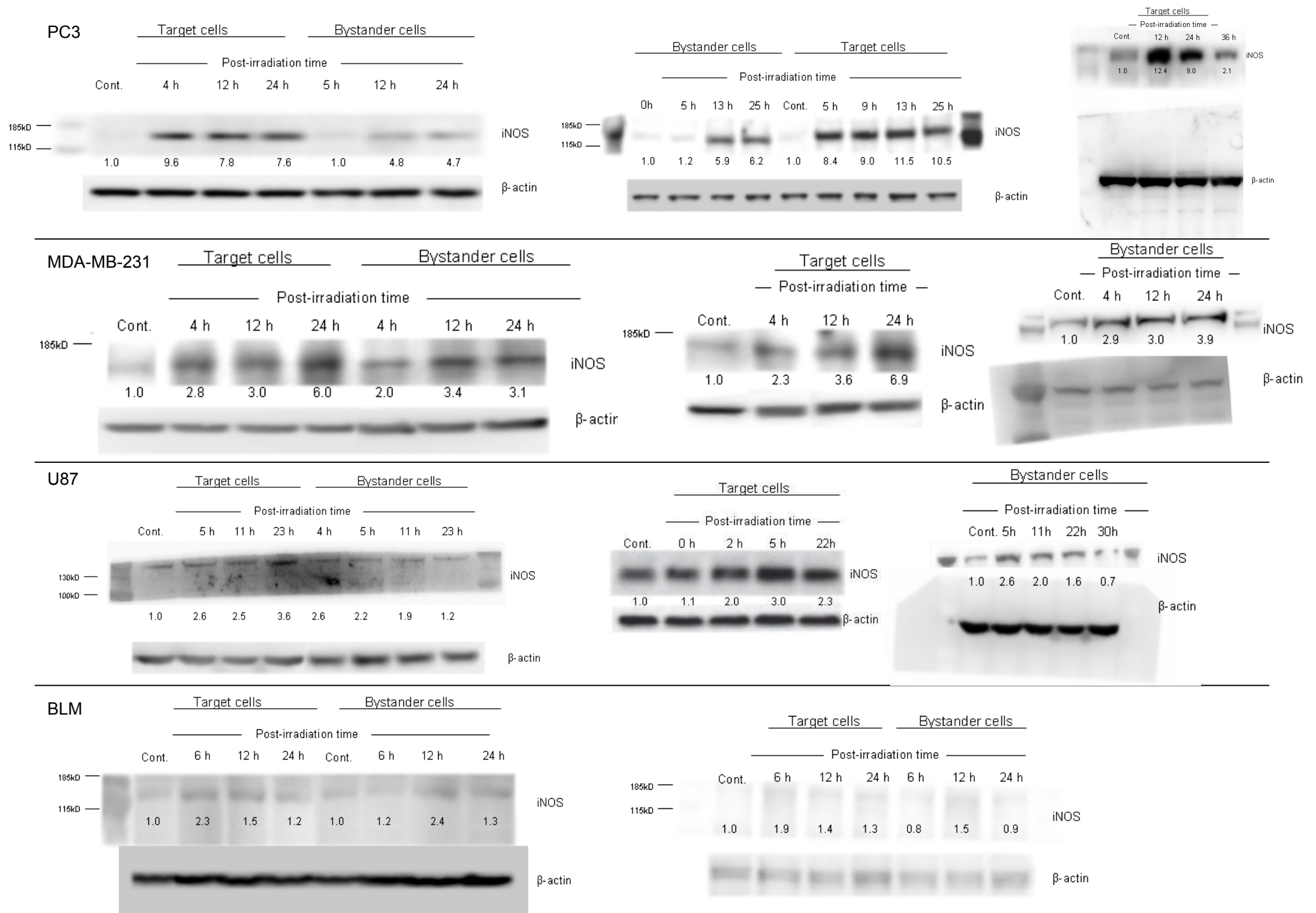

Supplement: Supplementary file 1 [file cancers-11-01674-s001.zip › supplementary-proof/WB supplement-proof.pdf]
